# Application of Cornelian Cherry (*Cornus mas* L.) Peel in Probiotic Ice Cream: Functionality and Viability during Storage

**DOI:** 10.3390/antiox10111777

**Published:** 2021-11-06

**Authors:** Shaghayegh Haghani, Milad Hadidi, Shiva Pouramin, Fateme Adinepour, Zahra Hasiri, Andrés Moreno, Paulo E. S. Munekata, José M. Lorenzo

**Affiliations:** 1 Department of Food Science and Industries, Khazar Institute of Higher Education, Mahmoudabad 86414-46318, Mazandaran, Iran; S.haghani@khazar.ac.ir (S.H.); S.pourmain@khazar.ac.ir (S.P.); 2 Department of Organic Chemistry, Faculty of Chemical Sciences and Technologies, University of Castilla-La Mancha, 13071 Ciudad Real, Spain; andres.moreno@uclm.es; 3 Department of Food Materials and Process Design Engineering, Gorgan University of Agricultural Sciences and Natural Resources, Gorgán 49138-15739, Golestan, Iran; F.adinepour@gau.ac.ir; 4 College of Veterinary Medicine, Islamic Azad University of Shahrekord, Shahrekord 88137-33395, Chaharmahal and Bakhtiari Province, Iran; Zahra.Hasiri@iau.ac.ir; 5 Centro Tecnológico de la Carne de Galicia, Rúa Galicia Nº 4, Parque Tecnológico de Galicia, San Cibrao das Viñas, 32900 Ourense, Spain; jmlorenzo@ceteca.net; 6 Área de Tecnología de los Alimentos, Facultad de Ciencias de Ourense, Universidad de Vigo, 32004 Ourense, Spain

**Keywords:** bioactive compound, by-product, antioxidant activity, functional food, total polyphenol, probiotic, dairy products, textural properties

## Abstract

In this study, cornelian cherry (*Cornus mas* L.) peel (CCP) was incorporated into a probiotic ice cream formulation containing *Bifidobacterium lactis* to investigate the potential effect of CCP on the viability of *B. lactis* in the ice cream after simulated gastrointestinal stress and during 120 days of storage. Furthermore, the effect of the addition of CCP (3, 6, and 9%) on bioactive compounds, antioxidant activity, and physicochemical and sensory attributes of the ice cream was evaluated. The results showed that the addition of CCP significantly enhanced vitamin C, total polyphenols, total anthocyanin content, and antioxidant activity of the ice cream. During frozen storage of the ice cream, phenolic compounds and anthocyanins were quite stable, but vitamin C significantly decreased. The addition of CCP had no significant effect on the viability of *B. lactis* throughout the freezing process, but increments of 6% and 9% CCP increased the viability of *B. lactis* in the ice cream and after simulated gastrointestinal processes in all storage periods. These findings imply that CCP is a promising candidate to be used for producing functional ice cream.

## 1. Introduction

Ice cream is one of the world’s leading dairy products. It provides vast amounts of calories to users as a frozen dairy dessert with plenty of nutrients [1]. Since it is a food with high nutritional value, valuable proteins, and significant levels of fat, it could be a suitable carrier of probiotic strains. On the contrary, ice cream is not rich enough in antioxidants such as phenolic compounds, dietary fiber, minerals, or vitamins [2].

Today, consumers have shown interest in the consumption of functional foods that contain more natural bioactive substances, probiotics, prebiotics, natural flavors, and colorants [3]. As a food component, probiotics are composed of live microorganisms (bacteria and/or yeasts) that induce beneficial effects in humans and animals mainly by balancing the intestinal flora [4]. The most common microorganisms used as probiotics belong to the genera of *Bifidobacterium* and *Lactobacillus*. *Bifidobacterium lactis* is a probiotic strain of human origin with well-documented beneficial health effects. It promotes the defense against intestinal pathogens and has been consumed largely since 1990 in food products [5]. Probiotic counts between 10^6^ to 10^9^ CFU/g are globally accepted in food [6,7]. Incorporating probiotics into ice cream during processing is a great challenge due to the complexity of maintaining probiotic viability, sensory acceptance, and the need for lower overrun [8].

Nowadays, fruit juice industries generate high quantities of by-products and wastes that contain high value-added ingredients with high functionality. Fruit peels are excellent natural sources of phenolic compounds, minerals, vitamins, and fibers, which are important to maintain health status [9,10]. Among fruits, cornelian cherry has currently gained increasing importance. It is an extremely rich source of phenolic compounds, anthocyanins, and vitamins that can be used as food ingredients [11]. Data in the literature have shown various biological functions related to phenolic compounds. Indeed, most of these biological properties have been ascribed to their potential antioxidant activity, as well as antimicrobial, antidiabetic, and anti-allergic activities [12]. To obtain these health benefits, they must reach the blood circulation after absorption by the intestinal wall. Moreover, during the simulated gastrointestinal process, phenolic compounds also have indicated protective effects on the viability of probiotics that can reduce the damage to probiotic cells when exposed to the rough conditions of gastrointestinal digestion [6]. Therefore, the use of cornelian cherry peel (CCP) as a natural source of bioactive ingredients seems advantageous to protect probiotic bacteria during digestion and generate a healthier ice cream with functional characteristics.

The aim of this investigation was to develop and improve the nutritional value of ice cream using CCP and *Bifidobacterium lactis*. This research assessed the effect of incorporating different concentrations of CCP on the proximate composition, physicochemical and antioxidant activity, and sensory attributes of probiotic ice cream. The survivability of probiotics after freezing and throughout 120 days of storage was determined. Moreover, the protective effect of CCP on the stability and viability of *B. lactis* to simulated gastrointestinal stress was also investigated.

## 2. Materials and Methods

### 2.1. Materials

Pasteurized skim cow’s milk powder, UHT milk, and cream of milk (30% fat content) were purchased from Letona Dairy Co., (Barcelona, Spain). Cornelian cherry fruit, stabilizer (salep), and white sugar were obtained from a local supermarket (Gorgan, Golestan, Iran). The chemical composition of CCP is presented in Table 1. Emulsifier (mono-diglycerides-E471) was purchased from Molteck Co., (Berlin, Germany). All chemicals were of food or analytical grade and were acquired from Sigma-Aldrich Ltd. (St. Louis, MO, USA) or Panreac Química S.A. (Barcelona, Spain). *Bifidobacterium lactis* (Bl-04) strain was obtained from DuPont™ Danisco^®^ (Barcelona, Spain).

### 2.2. Preparation of Ice Creams

Ice creams were produced in the Pilot Plant of the Good Food Institute (Gorgan, Iran). The mixes of ice cream were made by adjusting the cow’s milk fat ratio to 6% (*v*/*v*) with the addition of cream (30% fat). Afterward, 20 kg of the milk was divided into 4 equal portions. For each mix, 5% skim cow’s milk powder, 15% sugar, 0.75% stabilizer (salep) and 0.25% emulsifier (mono-diglycerides-E471) were added. The mixes were pasteurized using the HTST technique at 85 °C for 25 s in a plate heat exchanger and were quickly cooled to 2 °C. For incorporation of probiotic, *B. lactis* culture was previously dissolved into UHT milk and activated to obtain 10^9^ CFU/mL of bacteria cells in MRS broth. Then, it was added (1%, *v*/*v*) to the ice cream mix after pasteurization. Cornelian cherries were washed in distilled water and their seeds and peels were manually separated. Cornelian cherry peel (CCP) was ground by utilizing a Juice Fountain Juicer (BJE200XL, Breville co, Sydney, Australia) to obtain a fine puree. Later on, the CCP was added at three different concentrations (3, 6, and 9%) into the ice cream mix. The probiotic mix without CCP was considered as the control sample. The whipping-freezing process was applied with an ice cream maker at−6 °C (BCI600XL, Breville Co., Sydney, Australia), and the ice creams were hardened at −23 °C for 24 h and kept at −18 °C.

### 2.3. Analysis of Proximate Composition and Mineral Content

Total solid content by gravimetric method, protein content by Kjeldahl method, fat content by Gerber method, moisture, and ash content of the CCP and ice cream samples were estimated following AOAC methods [13]. A microwave oven (Berghof GmbH, Eningen, Germany) was employed to disrupt ice cream samples. An inductively coupled plasma spectrophotometer (GENESIS ICP-OES, SPECTRO Analytical Instruments, Kleve, Germany) was used to measure mineral content (Ca, Mg, P, K, and S) in the ice cream samples [14].

### 2.4. Physicochemical Properties of Ice Cream

Samples of hardened ice cream were melted in a refrigerator at 4 °C for a day, and then the refrigerator was set to reach 20 °C. A Brookfield viscometer (Dv2trv Brookfield, Stoughton, USA) with an RV spindle set (No. 2) was employed to determine the viscosity of melted ice creams. Melted ice cream samples (10 g) were mixed with distilled water (90 mL). The pH was measured with a pH meter (D-125, Corning Inc., New York, NY, USA) after homogenization. Meltdown behavior was determined following the methodology defined by Martinou-Voulasiki and Zerfiridis [15] with some modifications. Frozen ice creams (100 g) were stored in a freezer at −22 °C for 20 h, and the ice creams were subsequently put on a wire mesh and permitted to melt at 22 °C. Ice creams melted through the wire mesh were weighed every 10 min for 150 min.

For overrun measurement, a container (300 mL) was filled with the ice cream mix and weighed, and the same volume of hardened ice cream was cut and placed in a container with the same volume capacity. The overrun of ice creams (%) was calculated by applying Equation (1) as presented below:

Overrun (%) = (Weight of mix (g) − Weight of ice cream (g))/(Weight of ice cream (g)) × 100(1)

The firmness and cohesiveness of the ice creams were determined by a texture analyzer (CT3-100, Paul N. Gardner Company, Inc., Tampa, FL, USA) with a 50 mm cylinder probe. Frozen ice creams were cut into cubes (2 × 2 × 2 cm³) and were pressed at the speed of 5 mm/s. A Minolta colorimeter (Chroma Meter CR 410, Japan) was employed to measure the color of samples in the three-dimensional CIE L* a* b* system [14].

### 2.5. Determination of Bioactive Compounds 

The ascorbic acid content of the ice creams was measured with a reflectometer (RQflex plus 10, Merck, Darmstadt, Germany) and an ascorbic acid tester (EMD Millipore Reflectoquant 117963, Merck, Darmstadt, Germany). Ice creams were melted, homogenized for 1 min, and filtered prior to quantification [16].

For phenolic content determination, 25 mL of methanol/water solution (1:1) was used for extracting 25 g of melted ice cream for 12 h. The application of the methanol/water solution was able to extract both water and methanol extractable phenolic compounds. The solution was filtered using a Whatman paper (no. 4) in a 10 mL tube; afterwards, 0.2 mL of the sample was added to 1.8 mL distilled water. Folin–Ciocalteu’s phenol reagent (1 mL) was mixed with 2 mL Na_2_CO_3_ solution (20% *w*/*v*). The blend was maintained at room temperature for 20 min, and then the absorbance was recorded at 735 nm [2]. Gallic acid in a concentration range from 0 to 1 mg/mL was employed for the standard calibration curve, and the total phenolic content was expressed as mg gallic acid per mL of ice cream.

The method of Hwang, Shyu, and Hsu [2] with some modifications was used to determine the total anthocyanin content of the ice cream. Firstly, melted ice cream (2 mL) was dissolved in 2 mL methanol (including 10% HCl) and centrifuged at 3000× *g* for 10 min. The supernatant (1 mL) was mixed with 1 mol equiv/l HCl (9 mL). The absorbance was determined at 520 nm. The content of total anthocyanin was quantified as the absorbance at 520 nm × 101 × 18.89 (mg/L). The limit of detection (LOD) and limit of quantification (LOQ) concentrations were set using equations, where σ is the standard deviation of the y-intercept and S is the slope of the calibration curve: LOD = 3.3 × σ/S, LOQ = 10 × σ/S.

### 2.6. Determination of Antioxidant Activity

First, 20 g of ice cream was extracted with a 10 mL methanol/water solution (1:1) for 12 h. The blend was filtered with Whatman No. 4 paper. DPPH reagent (0.1 mM) was prepared in methanol, then 3.8 mL of this reagent was added to 100 μL of the extract, vortexed in test tubes for 15 s, and followed by incubation of the tubes in the dark for 24 h at room temperature. The color reduction was measured at 515 nm with a spectrophotometer [1]. The DPPH radical scavenging (%) was estimated according to Equation (2):

DPPH radical scavenging (%) = A0 − (A − Ab)/A0 × 100(2)

where A0 is absorption of the control (without sample), A is absorption of the sample with DPPH, and Ab is absorption of the sample without DPPH.

### 2.7. Gastrointestinal Resistance of Probiotic Ice Cream

Briefly, 1 g of ice cream sample was homogenized with 9 mL of sterile electrolyte solution (6.2 g/L NaCl, 2.2 g/L KCl, 1.2 g/L NaHCO_3_, 0.22 g/L CaCl_2_, and lysozyme 0.01%) in a Stomacher blender for 3 min. Then, 10 mL of electrolyte mixture including 0.3% pepsin was added to the solution. The pH was set to 2.0 by HCl solution (1.0 mol/L), and this mixture was incubated at 37 °C for 1 h. In the intestinal phase, the pH was increased to 7.5 with a sterile alkaline solution including bile (10 g/L) and pancreatin (1 g/L). Finally, the mixture was incubated under anaerobic conditions at 37 °C for 4 h [17].

### 2.8. Viability of B. lactis

The counts of *B. lactis* in the ice cream mix, frozen ice creams, and after simulated gastrointestinal stress were evaluated in triplicates during 120 days of frozen storage. For the enumeration of viable cell count, 10 g of ice cream was diluted and homogenized in 90 mL of peptone water. Then, serial decimal dilutions were subsequently prepared, and 0.1 mL of the mixture was inoculated on Petri plates including Man Rogosa Sharpe (MRS) agar by the pour plating technique. The plates were incubated anaerobically for 48 h at 37 °C. The results were expressed as log CFU/g [17].

### 2.9. Sensory Analysis

The sensory attributes of the ice creams were evaluated by 50 semi-trained panelists. Frozen ice creams were evaluated at a serving temperature of −10 °C. Three-digit random codes were assigned to samples, which were assessed regarding color, taste, odor, texture, and overall acceptability based on a 9-point hedonic scale (9 = like extremely and 1 = dislike extremely). Drinking water was supplied to the panelists for rinsing during evaluation.

### 2.10. Statistical Analysis

Statistical analysis was performed using SPSS software 23 (IBM Corporation, Somers, NY). Data were expressed as mean values and standard deviations (*n* = 3), which were analyzed by ANOVA and compared by the Duncan test. Differences were considered to be significant for values of *p* < 0.05.

## 3. Results and Discussion

### 3.1. Proximate Composition and Mineral Content of Ice Cream

As can be seen from Table 2, the incorporation of CCP significantly influenced the proximate composition of the ice creams (*p* < 0.05). The total solid (33.4–36.5%) and ash (0.96–1.42%) content of ice cream increased significantly with the increase in the levels of CCP (*p* < 0.05). Conversely, the addition of CCP to probiotic ice creams caused a decrease in protein (4.93 to 3.96%) and fat (5.95 to 4.49%) content. The Ca content of the probiotic ice creams was decreased (from 2481 to 2019 mg/kg) by the augmentation with CCP (Table 2). Cornelian cherry fruit is rich in micro and macro elements and can be a key source of essential nutrients for food fortification [18]. The highest levels of Mg and K were found in the ice cream supplemented with 6% CCP. Magnesium is an essential factor in the metabolism of bones. Its insufficiency has been outlined as a potential risk factor for osteoporosis. Potassium is necessary for many benefits to the human body and all living cells. Increasing the values of K in the diet can protect against hypertension in people who are sensitive to high levels of Na (*p* < 0.05) [19]. The Na content in ice creams decreased with the increment of CCP, but this decline was not statistically significant (*p* > 0.05).

### 3.2. Physicochemical Properties of Ice Creams 

Table 3 lists the results of the evaluation of physicochemical properties of the probiotic ice creams supplemented with different concentrations of CCP. The overrun of ice creams declined with the increase in CCP concentration. The low level of overrun was due to the low air temperatures in the ice cream and the cold impression occurring in the mouth. The importance of evaluating overrun is supported by the effect of this characteristic on the quality of the product, profit, and also compliance with the legal standard [20].

Viscosity is one of the most important factors in ice cream quality and depends on the ice cream structure. High viscosity causes hardening of the cream in the mouth and adhesion on the palate [21]. The viscosity of ice creams was significantly increased by incorporating CCP so that the highest viscosity value was obtained from samples containing 9% CCP (771.5 cP) and the lowest from the control sample (414.1 cP). Consequently, high viscosity reduced both air incorporation in the mixture and overrun. Similar results were observed with Cape gooseberry added into ice cream by Erkaya et al. [14].

A decrease in the pH value was found when the concentration of CCP was increased. Basically, the pH of ice cream was between 6.3 and 6.5. It is also the most important parameter and depends on the protein in the milk, dissolved gases, and mineral salts. The reduction in pH can be ascribed to the presence of different acid compounds in cornelian cherry, such as ascorbic acid and phenolic substances [22]. Sharma et al. [23] reported a pH reduction (6.79–5.71) in ice cream with the incorporation of fine wine lees. Oxidation of ethanol also caused a decrease in the pH of ice cream. The results indicated that the increment of CCP led to a decline in the lightness and yellowness of the ice cream, but increased redness. The L* value of ice creams supplemented with CCP was also lower than the control sample (*p* < 0.05). Additionally, the b* values of samples decreased significantly, from 1.43 to −4.20. In contrast, the a* values in all samples were increased by increasing the level of CCP.

**Figure 1:**
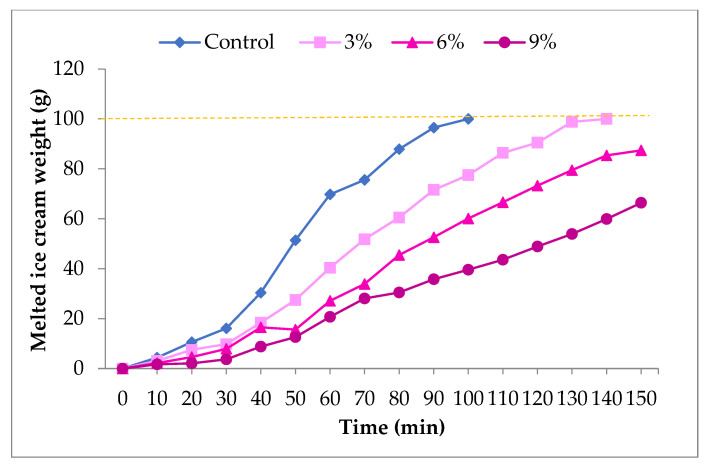
Melting behavior of probiotic ice creams supplemented with CCP.

In addition to overrun, the ice cream melting rate is also a significant quality index. Figure 1 indicates that after 100 min, the control sample was fully melted. The melting rate declined significantly due to CCP supplementation. After 150 min, the samples with 6% and 9% of CCP were melted (87 and 66 gr/100 g of ice cream, respectively). The melting rate of frozen dairy products is affected by several factors such as viscosity, hardness, ice crystal size, low overrun, total solids, and fat globule size. There was a difference in the melting behavior of ice cream samples due to the difference in their freezing point and viscosity [24]. Hence, it is suspected that cornelian cherry contains some ingredients that have water absorption ability (such as pectin), which could enhance melting resistance and viscosity. This hypothesis is supported by the study carried out by Jaćimović et al. [25], who reported a high amount of pectin in cornelian cherry fruits, making them highly recommended for different types of processing.

Firmness values of the probiotic ice creams varied from 182.5 to 322.6 g. Although CCP considerably reduced the ice cream overrun at 6% and 9%, it did not significantly change the firmness of the ice cream at 3%. The addition of CCP decreased the cohesiveness of the ice creams. Texture profiles were in agreement with the findings of Hwang et al. [2]. These authors indicated that the firmness and cohesiveness of ice cream were reduced due to an increase in the concentration of grape wine lees.

### 3.3. Bioactive and Antioxidant Activity of Ice Creams during Storage

Bioactive compound content and antioxidant activity of probiotic ice cream mixes (0 day), after freezing (first day) and during storage are listed in Table 4. The vitamin C was not found in the control. However, the highest mean vitamin C content was detected in 9% CCP ice cream mix, followed by the 6% and 3% CCP ice cream mixes. The freezing process and storage of samples significantly reduced the vitamin C content (*p* < 0.05). Pantelidis et al. [11] detected high vitamin C content (103 mg/100 g) in cornelian cherry fruits. Furthermore, these authors highlighted that the ascorbic acid content in cornelian cherry was remarkably higher than in other fruits such as strawberry (46 mg/100 g) and orange (31 mg/100 g).

The results showed that the increment of CCP significantly improved the total phenolic content in ice cream. Phenolic compounds are secondary metabolites and can be found in fruits and vegetables [26]. Enrichment of ice cream by some natural ingredients such as grape seeds [27], blackthorn [28], kiwifruit [20] and grape juice residue [29] increased total phenolic content and antioxidant capacity. Boris et al. [30] reported total phenolic content of cornelian cherries in the range from 494 to 704 mg gallic acid/100 g dry weight.

In addition, it did not decrease the total polyphenol content because of the freezing of ice cream and storage. For the ice cream control sample, the total anthocyanin content was not found; however, the anthocyanin level ranged from 0 to 0.42 mg/L and was increased significantly by adding CCP to the ice cream (*p* < 0.05). The cornelian cherry fruits have been indicated as the highest anthocyanin source among different small fruits (223 mg/100 g fw) [11]. An increase in anthocyanin content (11.5–28%) was observed in probiotic ice creams supplemented by CCP during the freezing process. The freezing process seems to increase anthocyanin content, which can be explained by the cellular breakdown and eventual release of compounds [31]. Moreover, it was indicated in another study that during the different processes of ice cream manufacture, anthocyanins and polyphenols were completely stable [2]. The sensitivity of the method was evaluated by determining LOD and LOQ. The LOD and LOQ for anthocyanins were 0.006 and 0.018 µg/mL, respectively. However, quantitative analysis of anthocyanin by a spectrophotometric method in complex mixtures has limitations due to the interaction of other red-colored compounds with anthocyanins.

The trends for the results obtained from the DPPH radical scavenging assay (Table 4) were similar to those for total phenolic and total anthocyanin content. Free DPPH radical scavenging showed a significant increase with the increment of the CCP concentration in probiotic ice cream. Sagdic et al. [32] implied that there was a strong correlation between free radical scavenging activity and the total phenolic content of ice cream. When adding 9% of CCP, the DPPH radical scavenging reached 70.5%. Hence, adding CCP may improve the antioxidant properties of probiotic ice creams. So, the high level of antioxidant activity in ice cream increases health benefits and also reduces the risk of many diseases. Cruxen et al. [33] reported that there was an increase in free radical scavenging activity by increasing the Butiá fruit puree content in ice cream. Similarly, Sagdic et al. [32] found that antioxidant activity in ice creams with added grape seed extract increased during 60-day storage. According to the authors, the presence of bioactive compounds including ascorbic acid, carotenoids, and phenolic compounds in fruits or their by-products, could be responsible for the improvement in antioxidant activity. Furthermore, Akca and Akpinar [34] stated that increasing the acidity of the fruit can increase the DPPH radical scavenging activity. It can be thought that the acidity of probiotic ice creams causes an increase in the DPPH radical scavenging of the CCP in ice cream.

### 3.4. Viability of B. lactis after Simulated Gastrointestinal Stress and during Storage

Table 5 shows *B. lactis* counts in ice cream mixes and frozen ice creams supplemented by CCP during storage and after simulated passage through the gastrointestinal system. The population of *B. lactis* decreased by 0.94–1.26 log cycles throughout the freezing process. The oxygen is toxic for most probiotic strains, so air incorporation into ice cream throughout freezing may explain the decrease in *B. lactis* counts. Additionally, the reduction in the probiotic population during the freezing process may result from thermal shock and injuries to bacterial cells [6]. The addition of CCP had no significant effect on the viability of *B. lactis* during the freezing process, but the increment of 6% and 9% CCP in ice cream increased the viability of *B. lactis* during 120-day storage and after gastrointestinal simulation. Öztürk, Demirci and Akin [1] reported that the addition of blue and white fruits of *Myrtus communis* augmented the survival of *Lactobacillus casei* in the ice cream during 8 weeks of storage.

Likewise, the ice cream formulated with Butiá fruit had cryoprotective characteristics that enhanced the viability of *B. Lactis* during storage [33]. Costa et al. [35] observed that *Lb. rhamnosus GG* added in Amazonian palm berry ice cream exhibited higher viability under simulated gastrointestinal conditions compared to the fresh culture. In this sense, the presence of the food matrix contributed to the probiotic’s viability. In this regard, the high level of bioactive compounds in CCP (such as phenolic compounds, dietary fibers, vitamins, and minerals) may have even assisted probiotic viability. The survival of *B. lactis* under in vitro simulated gastrointestinal conditions decreased significantly during storage. However, in ice creams supplemented by 6% and 9% CCP and stored for 120 days at −18 °C, the counts were higher than 10^6^ CFU/g, which indicates a synbiotic ice cream. Balthazar et al. [36] found a reduction of about 2 log cycles for the viability of bacteria after simulated gastrointestinal stress in synbiotic sheep’s milk ice cream after 150 days of storage.

### 3.5. Sensory Analysis of Ice Cream

**Figure 2:**
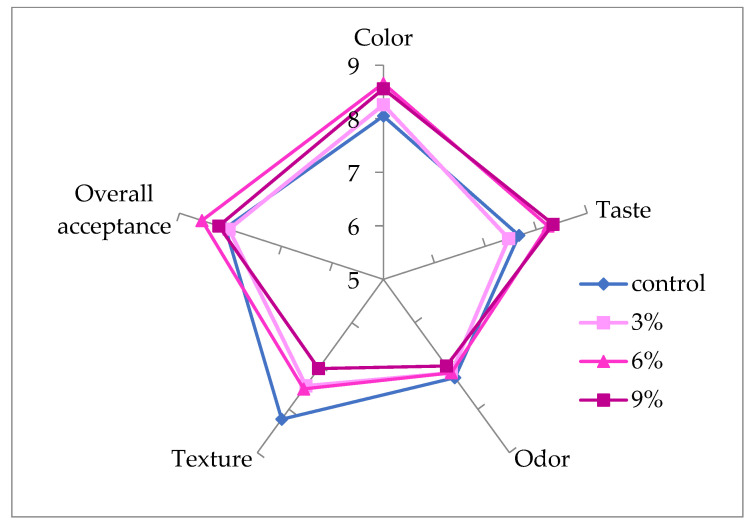
Sensory evaluation of probiotic ice creams supplemented with CCP.

As seen in Figure 2, the incorporation of CCP into the ice cream significantly (*p* < 0.05) affected sensory acceptability. Although the increment of CCP had no significant effect (*p* > 0.05) on the odor, the scores for color and taste of the probiotic ice cream improved with the increase in CCP concentration. These attributes of probiotic ice cream with 6% and 9% CCP were statistically different from the other samples (*p* > 0.05). The lowest texture scores were found to be 7.06 in the sample with 9% CCP. The highest overall acceptance scores were observed in ice cream containing 6% CCP, which was followed by 9%, 3% and control samples, respectively. Karaman et al. [37] also employed hedonic scales to investigate ice cream supplemented with persimmon puree at different levels. These authors indicated that concentrations higher than 24% decreased the overall acceptance score. In the present study, the general acceptability scores of probiotic ice cream with 3% CCP were similar to the control sample. Consequently, the addition of CCP caused an increase in the overall sensory properties. As a result, an ice cream including 6% CCP was the mix most highly preferred by the panelists.

## 4. Conclusions

Fruit by-products are gaining great importance for our world today. Fruit peel wastes are used as nutritional additives and structure regulators in many areas of industry. It is very important in terms of by-product evaluation that the by-products remaining after the production of fruit juice can be reutilized in foods, as they contain phenolic and antioxidant components. Ice cream is poor in phenolic substances. Therefore, the present study was carried out to improve the functional properties of ice cream by adding cornelian cherry peel as a by-product of the juice industry. In this study, a functional ice cream containing different concentrations of CCP and *B. lactis* was developed, and it was indicated that using CCP can improve the viscosity, antioxidant activity, and nutritive value of ice cream. In addition, due to the presence of polyphenol compounds, the survival of *B. lactis* increased during 120 days of storage and in simulated gastrointestinal conditions. It can be concluded that CCP may be considered as a functional ingredient in the formulation of synbiotic ice cream while maintaining sensory acceptability and probiotic viability.

## Figures and Tables

**Table 1:** Chemical composition of CCP.

Compounds	Level (Unit)
Total soluble solids	14.8 ± 0.3%
Fat	0.28 ± 0.01%
Protein	2.46 ± 0.08%
Ash	1.08 ± 0.02%
Vitamin C	97.43 ± 4.19 mg/100 g
Total phenolic content	638.2 ± 17.5 mg gallic acid/100 g

**Table 2:** Chemical and mineral composition of probiotic ice creams supplemented with CCP.

Component	Treatments			
	Control	3%	6%	9%
Total solid (%)	33.4 ± 0.6 ^c^	34.6 ± 0.6 ^b^	35.7 ± 0.5 ^b^	36.5 ± 0.7 ^a^
Fat (%)	5.95 ± 0.1 ^a^	5.55 ± 0.2 ^b^	4.96 ± 0.7 ^c^	4.49 ± 0.1 ^d^
Protein (%)	4.93 ± 0.5 ^a^	4.64 ± 0.1 ^a^	4.15 ±0.2 ^b^	3.96 ± 0.3 ^b^
Ash (%)	0.96 ± 0.1 ^b^	1.12 ± 0.1 ^b^	1.34 ± 0.1 ^a^	1.42 ± 0.2 ^a^
Ca (mg/kg)	2481.2 ± 21 ^a^	2352.7 ± 18 ^b^	2211.5 ± 25 ^c^	2019 ± 14 ^d^
Mg (mg/kg)	315.8± 11 ^c^	398.8 ± 14 ^b^	437.4 ± 12 ^ab^	478.2 ± 9 ^a^
P (mg/kg)	1018.8 ± 25 ^a^	901.3 ± 18 ^ab^	779.3 ± 38 ^b^	624.2 ± 18 ^c^
K (mg/kg)	2321.4± 37 ^d^	2547.2 ± 28 ^c^	2787 ± 20 ^b^	3013.1 ± 33 ^a^
Na (mg/kg)	670.3 ± 35 ^a^	654.1 ± 18 ^a^	649.3 ± 33 ^a^	630 ± 24 ^a^

Mean values (*n* = 3) with different lower-case letters in the same row are significantly different (*p* < 0.05).

**Table 3:** Physicochemical properties of probiotic ice creams supplemented with CCP.

Component	Treatments			
	Control	3%	6%	9%
Overrun (%)	56.56 ± 2.3 ^a^	53.53 ± 1.7 ^b^	49.13 ± 0.4 ^c^	47.46 ± 0.2 ^c^
Viscosity (cP)	414.1 ± 26.1 ^d^	567.3 ± 35.2 ^c^	656.3 ± 13.3 ^b^	771.5 ± 32.4 ^a^
Firmness (g)	322.6 ± 45.5 ^a^	297.8 ± 36.2 ^a^	235.3 ± 25.8 ^b^	182.5 ± 21.7 ^c^
Cohesiveness	0.28 ± 0.03 ^a^	0.26 ± 0.04 ^ab^	0.24 ± 0.02 ^b^	0.20 ± 0.01 ^c^
pH	6.8 ± 0.4 ^a^	6.6 ± 0.1 ^b^	6.2 ± 0.2 ^c^	5.7 ± 0.1 ^d^
L*	97.20 ± 1.7 ^a^	88.43 ± 2.4 ^b^	82.90 ± 3.1 ^c^	80.90 ± 2.6 ^c^
a*	0.36 ± 0.1 ^d^	18.73 ± 0.2 ^c^	23.40 ± 0.9 ^b^	27.6 ± 1.2 ^a^
b*	1.43 ± 0.4 ^d^	−2.46 ± 0.1 ^c^	−3.66 ± 0.1 ^b^	−4.20 ± 0.3 ^a^

Mean values followed by different letters in the same row are significantly different (*p* < 0.05).

**Table 4:** Bioactive compound content in the probiotic ice cream mixes, after freezing and during storage.

Samples	Days	Ascorbic Acid	Total Polyphenol	Total Anthocyanins	DPPH
		(mg/kg DM)	(mg/mL)	(mg/L)	(%)
Control	0	n.d	1.120 ± 0.047 ^e^	n.d	8.1 ± 0.5 ^i^
	1	n.d	1.023 ± 0.028 ^e^	n.d	8.3 ± 2.4 ^i^
	60	n.d	0.975 ±0.050 ^e^	n.d	8.1 ± 0.9 ^i^
	120	n.d	1.071 ± 0.079 ^e^	n.d	7.9 ± 1.3 ^i^
3%	0	7.93 ± 0.57 ^g^	3.349 ± 0.325 ^d^	0.097 ± 0.004 ^f^	24.8 ± 0.7 ^g^
	1	6.83 ± 0.81 ^h^	3.407 ± 0.101 ^d^	0.135 ± 0.016 ^e^	25.5 ± 1.2 ^g^
	60	6.26 ± 0.24 ^hi^	3.257 ± 0.264 ^d^	0.142 ± 0.011 ^e^	22.1 ± 0.8 ^h^
	120	5.79 ± 0.38 ^i^	3.224 ± 0.097 ^d^	0.140 ± 0.007 ^e^	21.9 ± 2.7 ^h^
6%	0	14.18 ± 0.85 ^d^	4.596 ± 0.091 ^c^	0.191 ± 0.025 ^d^	45.3 ± 1.5 ^d^
	1	13.07 ± 1.32 ^e^	4.686 ± 0.166 ^c^	0.216 ± 0.008 ^c^	45.7 ± 3.1 ^d^
	60	11.02 ± 1.87 ^f^	4.445 ± 0.207 ^c^	0.210 ± 0.013 ^c^	41.7 ± 1.2 ^e^
	120	10.85 ± 0.96 ^f^	4.298 ± 0.101 ^c^	0.203 ± 0.019 ^c^	37.7 ± 2.9 ^f^
9%	0	20.11 ± 1.76 ^a^	6.057 ± 0.220 ^a^	0.332 ± 0.019 ^b^	59.7 ± 2.2 ^a^
	1	17.74 ± 1.38 ^b^	6.139 ± 0.112 ^a^	0.405 ± 0.016 ^a^	60.5 ± 3.7 ^a^
	60	16.14 ± 0.84 ^c^	5.762 ± 0.319 ^ab^	0.427 ± 0.005 ^a^	54.5 ± 1.5 ^b^
	120	13.59 ± 2.09 ^de^	5.347 ± 0.095 ^b^	0.398 ± 0.021 ^a^	52.5 ± 0.7 ^c^

Mean values (*n* = 3) with different lower-case letters in the same column are significantly different (*p* < 0.05). Day 0 means ice cream mix before freezing, n.d: value was not detected.

**Table 5:** Viability of *Bifidobacterium lactis* in the ice creams and after simulated gastrointestinal digestion during storage.

Samples	Days	Viability (Log CFU/g)	
		In Ice Cream	After Simulated Gastrointestinal Digestion
Control	0	8.86 ± 0.16 ^a^	-
	1	7.62 ± 0.12 ^c^	6.10 ± 0.12 ^b^
	60	7.29 ± 0.10 ^de^	5.24 ± 0.12 ^d^
	120	7.16 ± 0.10 ^e^	5.32 ± 0.12 ^cd^
3%	0	8.81 ± 0.08 ^a^	-
	1	7.55 ± 0.11 ^c^	5.97 ± 0.11 ^b^
	60	7.42 ± 0.19 ^cd^	5.35 ± 0.11 ^cd^
	120	7.32 ± 0.11 ^de^	5.49 ± 0.11 ^c^
6%	0	8.95 ± 0.24 ^a^	-
	1	8.01 ± 0.22 ^b^	6.43 ± 0.22 ^a^
	60	7.55 ± 0.08 ^c^	6.04 ± 0.22 ^b^
	120	7.41 ± 0.05 ^cd^	6.13 ± 0.22 ^b^
9%	0	8.81 ± 0.19 ^a^	-
	1	7.89 ± 0.11 ^b^	6.31 ± 0.11 ^a^
	60	7.60 ± 0.27 ^c^	6.35 ± 0.11 ^a^
	120	7.45 ± 0.07 ^cd^	6.05 ± 0.11 ^b^

Mean values (*n* = 3) with different lower-case letters in the same column are significantly different (*p* < 0.05). Day 0 means ice cream mix before freezing.

## Data Availability

Data is contained within the article.
